# Effect of polyethylene terephthalate particles on filamentous bacteria involved in activated sludge bulking and improvement in sludge settleability

**DOI:** 10.1038/s41598-023-48257-4

**Published:** 2023-11-25

**Authors:** Su Wei, Ziyang Huang, Yongjiong Ni, Zengrui Pan, Hongbo Feng, Xiaoyu Cheng, Zuchao Huang, Hanglei Liao, Jun Li

**Affiliations:** 1 College of Civil Engineering, Zhejiang University of Technology, Hangzhou, 310014 China; 2 College of Environment, Zhejiang University of Technology, Hangzhou, 310014 China

**Keywords:** Biochemistry, Environmental sciences, Environmental social sciences

## Abstract

Excessive proliferation of filamentous bacteria within activated sludge leads to sludge structural instability and diminished settling properties, which is a prevalent issue in tannery wastewater treatment. Based on available information, there is a lack of research on the impact of particle addition on filamentous bacteria in activated sludge, specifically with respect to a reduction in sludge bulking. Therefore, polyethylene terephthalate (PET) was selected as the test material to elucidate the effect of particles on sludge bulking. The results illustrate that particles measuring 0.1 mm in diameter have a profound influence on both the quantity and morphological characteristics of filamentous bacteria in activated sludge. In an anaerobic-aoxic-oxic (AAO) reactor, the use of 4000 particles/L led to a significant decrease in the sludge volume index (SVI), reducing it from 358 mg/L to 198 mg/L. The results offer significant insights for resolving sludge bulking problems in tannery wastewaters. Moreover, the results are significant as a reference point for future investigations on the efficacy of incorporating diverse particulate materials to ameliorate issues associated with activated sludge bulking.

## Introduction

The application of the activated sludge process is extensive in wastewater treatment plants, included in over 90% of municipal wastewater treatment and approximately 50% of industrial wastewater treatment^1^. Due to the high prevalence and time-consuming nature of activated sludge bulking, it has persisted as a formidable challenge in the realm of operational management and control^2,3^. Filamentous bacterial expansion results in an increase in sludge volume due to excessive bacterial propagation within the activated sludge floc, and previous research has shown that it is the primary cause of activated sludge bulking, accounting for more than 90% of occurrences^4–6^. Typically, the growth rate of bacterial micelles is higher than that of filamentous bacteria, and this reduces the possibility of the excessive proliferation of filamentous bacteria. Nonetheless, when environmental conditions within an activated sludge system deteriorate, e.g., the temperature shifts from its typical range to lower or slightly elevated levels, filamentous bacteria, which have larger surface areas relative to bacterial flocs, exhibit a heightened capacity to adapt to these alterations^7,8^. Under such circumstances, the abundance of filamentous bacteria can exceed the population of bacterial flocs, leading to sludge bulking. Palm et al.^9^ demonstrated that a volume fraction of filamentous bacteria within a range of 1–20% can induce sludge bulking. Notably, Kaewpipat et al.^10^ suggested that it is possible for the concentration of filamentous bacteria to be below a detectable limit during sludge bulking. This suggests that even though filamentous bacteria are not the dominant flora in wastewater treatment plants, they can still cause sludge bulking^11^.

The activated sludge treatment process has favourable sedimentation performance, with a water content of approximately 99% during normal operation^12^. However, when sludge bulking occurs (total length of filamentous bacteria ≥ 104 m/g), the activated sludge structure becomes loose and expands, causing a rapid increase in the sludge volume index (SVI) and a subsequent decrease in sludge settling performance. This results in a reduced concentration of returned sludge and poses challenges for solid‒liquid separation within secondary sedimentation tanks^13,14^. In the middle and late stages, a large number of bubbles of biological origins are produced, and it is difficult to maintain normal biochemical treatment performance; when the SVI reaches more than 300 mL/g, activated sludge losses lead to a turbid effluent, a high content of suspended solids (SS), and poor effluent quality, which strongly affects the stable operation of wastewater treatment plants^15,16^. Mao et al.^17^ found that the sludge settling velocity (SV) in wastewater treatment plants where activated sludge bulking occurred reached more than 60%; if the SVI exceeded 200 mL/g, the wastewater treatment process almost completely stagnated. In addition, the large amount of bulked activated sludge present increased the maintenance and operation cost of sludge treatment equipment, which adversely affected the circular economy. Sludge bulking poses a significant challenge to the operation and management of wastewater treatment plants because it is inherently difficult to control and requires additional time to address. Therefore, it has become an urgent issue to be addressed within this field^18^.

Researchers have carried out many studies on the identification of sludge bulking to reduce incidences of sludge bulking. Wang et al.^19^ used fluorescence in situ hybridization (FISH) technology to optimize hybridization time and other aspects of FISH to address the low probe permeability and poor FISH results caused by the physiological characteristics of filamentous bacteria, and the accuracy of filamentous bacteria identification was improved. Scholars also proposed a series of methods to inhibit sludge bulking by exploring the characteristics and operation process of urban wastewater treatment. The addition of agents and changes in operating conditions (e.g., increasing aeration time to inhibit filamentous growth) to rapidly control filamentous growth in activated sludge are common solutions in current wastewater treatment plants^20,21^. Vijay et al.^22,23^ studied the composition of extracellular polymer substances (EPS) and the characteristics of microbial flocs. Their findings confirmed that in situ sludge minimization for industrial wastewater can be effectively carried out. This research is significant for enhancing our understanding of the behaviour of activated sludge. Peng et al.^24^ discovered that by maintaining typical operating and environmental conditions while specifically decreasing the level of dissolved oxygen, it is possible to mitigate the extent and rate of sludge bulking. In their study, Bai et al.^25^ proposed a modification to the anaerobic-oxic-oxic (AAO) process, replacing it with an anaerobic-oxic (AO) process. This modification is suggested as a means to effectively regulate the proliferation of filamentous bacteria.

In addition to sludge, large amounts of particles are found in municipal wastewater treatment plants in recent years^26^. To solve the carbon source problem, an increasing number of wastewater treatment plants have eliminated primary sedimentation tanks; as a result, more particles enter the biochemical tanks of wastewater treatment plants. Therefore, wastewater treatment plant effluent has a wide variety of particle sources, and most of these particles are difficult to degrade^27–30^. Current studies on the effect of particles on activated sludge are more focused on inorganic particles and less on other particles, and studies mainly assess sludge settleability^31^, activated sludge microbial activity^28^, and activated sludge granularity^32^. An exploration of the relationship between particles and sludge bulking is lacking^33^. Therefore, in this study, the effect of particles on filamentous bacteria and activated sludge bulking was analysed through the addition of PET particles of different sizes. The particles suppressed and reduced activated sludge bulking to some extent.

## Material and methods

### Materials

Polyethylene terephthalate (PET), one of the most widely occurring microplastics in wastewater treatment plant effluent^34–37^, was selected as the experimental particle. Polyethylene terephthalate particle raw materials were purchased from a plastic raw material company in Dongguan, China, and three different particle of sizes 0.075–0.15 mm (PET-1), 0.3–0.6 mm (PET-2), and 0.6–1.18 mm (PET-3) were obtained through screening, rinsing and drying. The density of the PET particles was found to be 1.38 g/cm^3^. Particle size was measured by a laser particle size analyser (Yishite, LAP-W2000H, China). Table 1 displays the three particle sizes for the PET.

**Table 1:** Particle size distribution of the different PET particles.

Sample number	Cumulative 10% particle size (μm)	Cumulative 50% particle size (μm)	Cumulative 97% particle size (μm)	Specific surface area (m^2^/kg)	Average particle mass (g/piece)
PET-1	73.73	111.61	152.65	72.79	0.769 × 10^–5^
PET-2	354.27	450.22	674.46	10.07	0.298 × 10^–3^
PET-3	641.97	936.49	1346.99	6.83	0.920 × 10^–3^

Activated sludge was taken from an oxidation pond of a wastewater treatment plant in Hangzhou. To minimize the influence of other particles in activated sludge, the sludge was screened with a metal screen with a diameter of 0.3 mm. The biological activity and sedimentation of sludge were deemed satisfactory, with the mixed liquid suspended solid (MLSS) and SVI measuring 5095 mg/L and 115 mL/g, respectively.

### Batch (shaking flask) experiment

The effect of PET particles on sludge bulked with filamentous bacteria was investigated in a comparative shaking bed experiment using 250 mL conical glass flasks, which was achieved using the same method as Liu et al.^38^. A volume of 100 mL of filament-bulked activated sludge was introduced into each conical flask. Subsequently, 2000 particles of PET-1, PET-2, and PET-3 were added to the flasks, with a blank for comparison. The conical flasks were placed in a thermostatic oscillator (Yiheng, THZ-98AB, China) for oscillation at a rate of 200 rpm and a temperature of 20 °C. The conical flasks underwent agitation for 24 h, with a 45-min pause in each cycle. The aim of this experimental procedure was to assess the rate of sedimentation of the sludge, observe the biological phase, and replenish the carbon source at a rate of 30% replacement. Bulked activated sludge was obtained from a laboratory AAO reactor with an MLSS of 2240 mg/L, SVI of 348 mL/g, and poor sludge settling performance, with a filamentous bacteria abundance of grade E. The filamentous bacteria were identified by natriuretic staining as predominantly natriuretic-negative bacteria, with straight mycelia, cells with diaphragms, and no adherent growth, and the predominant strain was Type 0803 bacteria.

### AAO continuous-flow experiment

**Figure 1:**
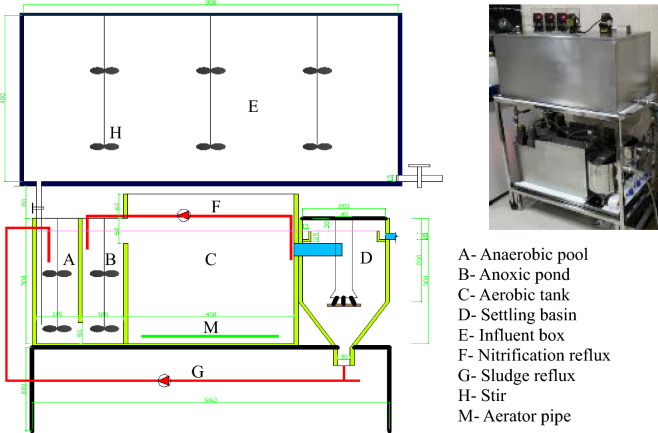
AAO reactor.

To study the effect of the addition of PET particles on sludge bulking, two sets of AAO experimental reactors with the same specifications were designed^39^. The AAO experimental reactor was made of stainless steel, with a total volume of 21.8 L, of which the volume ratio of the anaerobic tank, anoxic tank, aerobic tank, and vertical sedimentation tank was approximately 1:1:4:2 (as shown in Fig. 1). The effluent, nitration liquid, and sludge reflux of each AAO unit were individually controlled by a peristaltic pump. The pump pipe was made of a food-grade silicone tube, and the sludge remaining in the system was regularly discharged manually. The AAO reactor was operated in continuous flow mode with a treatment flow of 1.5 L/h. The dissolved oxygen content in the aerobic section was set to 2–4 mg/L, and that in the anoxic section was approximately 0.2 mg/L. The first stage of the reactor operation caused filamentous bacterial expansion in the activated sludge; the second stage was a comparative experiment in which one set of anaerobic tanks was supplemented with PET particles and the other set was not supplemented with any particles. The amount of PET particles to be added was determined by evaluating the results of the oscillation batch test (shown in Sect. “Batch (shaking flask) experiment”). The experiment was run continuously for 20 days after the addition of PET particles, and the quantity of PET particles in the effluent and the quality of influent and effluent, SV, and SVI of activated sludge were measured at the same time. It is worth mentioning that two AAO reactors were tested with sodium acetate, ammonium chloride, and potassium dihydrogen phosphate as influent feed, and the water quality is shown in Table 2.

**Table 2:** Water quality of influent.

COD (mg/L)	NH_3_-N (mg/L)	TP (mg/L)
900–1200	30–50	3–5

### Analytical methods

#### Sedimentation rate of activated sludge

A 100 mL glass cylinder was used to measure out 100 mL of deionized water. One drop of the activated sludge mixture was dropped onto the surface of the liquid in the measuring cylinder using a glass dropper with a plastic head. When the activated sludge settled to the 50 mL (88 mm) line on the scale, the settling time was recorded with a stopwatch, the rate of settling was calculated, and three parallel measurements were carried out.

#### Quantity of PET particles

A volume of 20–30 mL activated sludge was placed in a 100 mL beaker (the wall-attached sludge was rinsed with distilled or pure water and transferred), and then the beaker was placed in a 60 °C oven to dry. Polyethylene terephthalate particle analysis in the AAO reactor effluent was conducted after filtration. The effluent (1 L) was subjected to filtration using metal screens with mesh sizes of 150 and 500. The particles captured by the screens were then rinsed with pure water into a 100 mL beaker, with an approximate volume of 20 mL. For subsequent digestion and density separation, refer to the methods of Wei et al.^40^. In this study, a key distinction was the use of a separation solution containing 50% CaCl_2_, which has a higher density, approximately 1.5 g/cm^3^. Due to the known sizes of the PET particles used in this experiment, glass fibre filter paper 47 mm in diameter with 1.0 μm apertures (Whatman, GF/B1821-047, UK) was dried and placed directly under a stereomicroscope (Olympus, SZ61, Japan) for observation and counting.

#### Identification and evaluation of filamentous bacteria

Identification of dominant filamentous bacteria was conducted based on morphological observations and microbiological staining (Gram and Neisser) techniques as described in earlier works^41,42^. The filamentous index (FI), a method of subjective scoring of filamentous bacterial abundance suggested by Eikelboom^42^, was applied to evaluate the abundance of filamentous bacteria present in the samples.

#### Other methods

The sludge settling velocity (SV) was measured using a canning method. The mixed liquid suspended solid (MLSS) was obtained using a drying and weighing method. Other water quality indices, such as chemical oxygen demand (COD_Cr_), ammonia nitrogen (NH_3_-N), and total phosphorus (TP), were determined according to standard analysis methods (APHA, 2005)^43^.

## Results and discussion

### Extended filament control in the batch experiment

**Figure 2:**
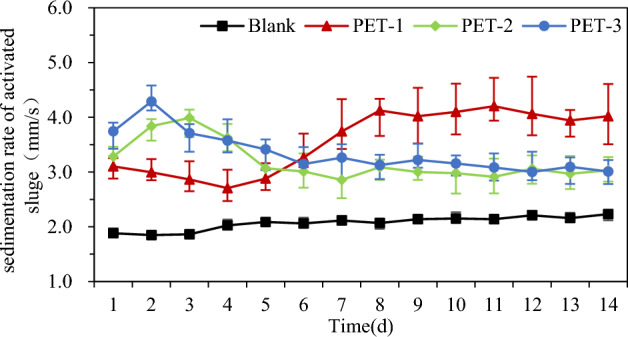
Effect of inert particles on the sedimentation rate of activated sludge on a shaking table.

Given the limited quantity of activated sludge utilized in the batch experiments, the sedimentation rate of the activated sludge was employed as an assessment metric to evaluate the impact of PET particles on sludge settleability. The results are shown in Fig. 2. It can be seen from the figure that the sedimentation rate of blank activated sludge was improved, but the effect was not obvious. After the addition of PET particles, the three kinds of particles increased the sedimentation rate, but their effect on the activated sludge sedimentation rate was different. The error in the PET-1 test was large, which may have been caused by the particles of size 0.1 mm, which easily adsorb to activated sludge, and by the inclusion of particles in the sludge.

Figure 2 shows a significant improvement in the settling rate within 24 h of the introduction of particles. It was also observed that the sedimentation rate was directly proportional to the size of the particles, indicating that larger particles resulted in a higher sedimentation rate. The sedimentation rate of the activated sludge supplemented with PET-3 and PET-2 particles reached its peak on Day 2 and Day 3, after which it began to gradually decrease. The sedimentation rate of activated sludge supplemented with PET-1 particles decreased steadily over 4 days before increasing significantly. After 6 days, the sedimentation rate of activated sludge containing PET-2 and PET-3 particles remained stable at 3 mm/s. While the effect of PET-1 particles on the sedimentation rate of activated sludge persisted, the sedimentation rate eventually reached 4 mm/s after 8 days. The impact and disruption effect of the particles on activated sludge varied due to the differing masses of the three types of particles, even when the sludge was subjected to the same rotation speed. The PET-3 particles have the largest size, followed by the PET-2 particles, while the PET-1 particles have the smallest size. The introduction of particles into bulked sludge results in a disruption and destruction of the initial activated sludge structure, primarily due to impact and disruption. It is worth noting that the degree of impact becomes more pronounced as the particle size and mass increase. Lin et al.^44^ found that PET particles have an enduring effect on activated sludge, leading to the inhibition of EPS. This inhibition negatively affects the flocculation process in activated sludge and compromises its settling performance. With PET-2 and PET-3 particles, the sedimentation rate of activated sludge exhibited a reduction of approximately 3 mm/s, which aligned with the established conclusions.

**Figure 3:**
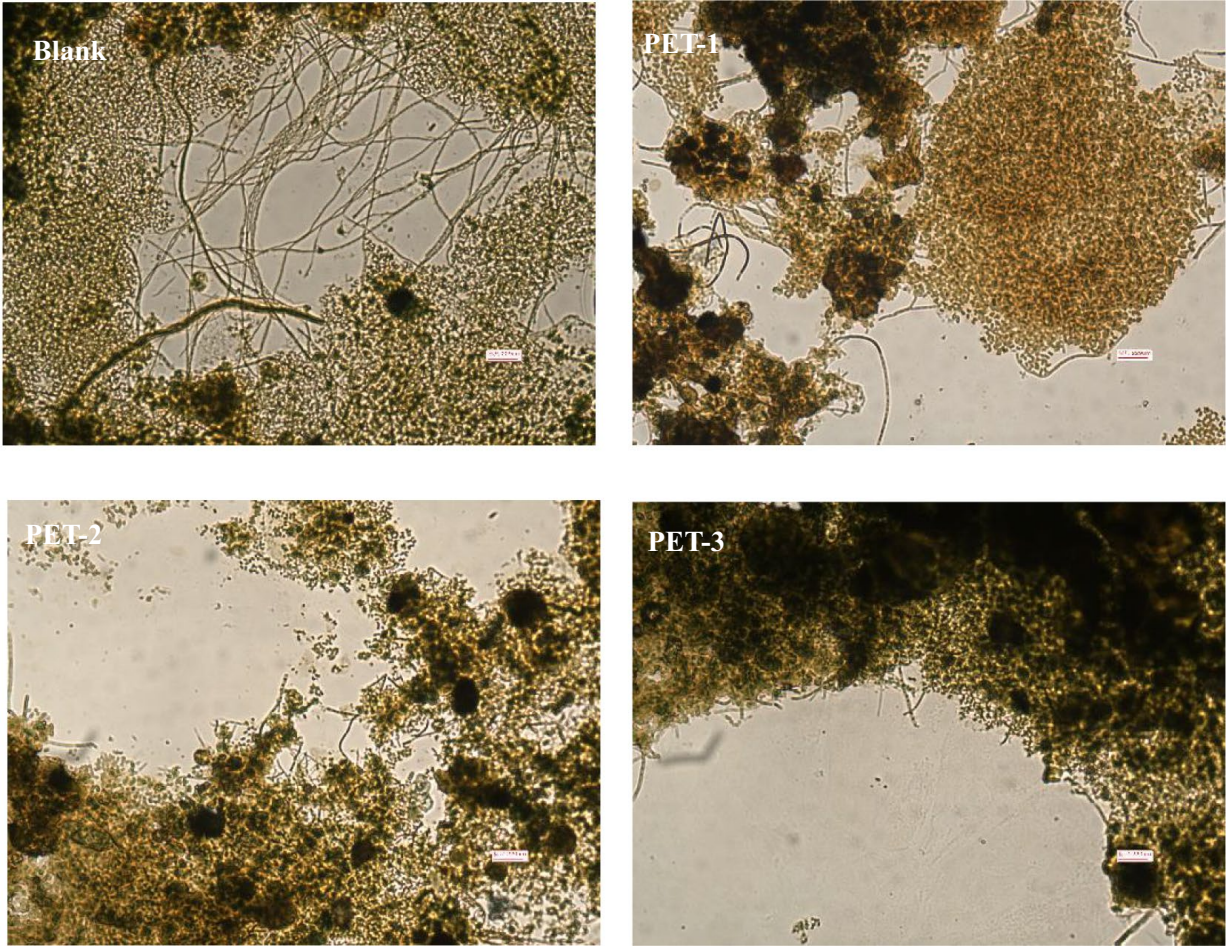
Effect of inert particles on filamentous bacteria in activated sludge.

Figure 3 shows images of the activated sludge subjected to the Neisser staining technique during the 6th day of the oscillating batch test. In comparison to the initial bulked sludge, oscillation and stirring resulted in a decrease in filamentous bacteria. However, the introduction of particles led to a notable reduction in both the density and length of filamentous bacteria compared to the control samples. The addition of PET-1 particles facilitated the growth of filamentous bacteria, whereas the presence of PET-2 and PET-3 particles resulted in a decrease in the abundance of filamentous bacteria within the activated sludge. Based on an examination of the growth pattern of filamentous bacteria, it was observed that the blank filamentous bacteria predominantly grew through the extension of activated sludge flocs. With PET-1 addition, the filamentous bacteria primarily grew intracellularly. The reduction in filamentous bacteria observed can be primarily attributed to the influence of particles, with the degree of reduction becoming more pronounced as the particle size increases.

The morphology of filamentous bacteria is closely tied to their genera, with common forms including straight, smooth, curved, spiral, and irregular shapes^13^. The original bulked sludge primarily exhibited straight filamentous bacterial forms. As illustrated in Fig. 3, when subjected to oscillating conditions, the blank samples showed a decrease in filamentous bacterial numbers, but the predominantly straight form of the bacteria was maintained. Figure 3 shows a comparison of filamentous bacterial morphology between the blank sample and the other three groups to which different particles were added and reveals distinct characteristics. Specifically, the filamentous bacteria displayed a more curved and inwards-growing morphology when PET-1 particles were added. In contrast, the filamentous bacteria in the other two particle groups appeared shorter and less abundant.

**Figure 4:**
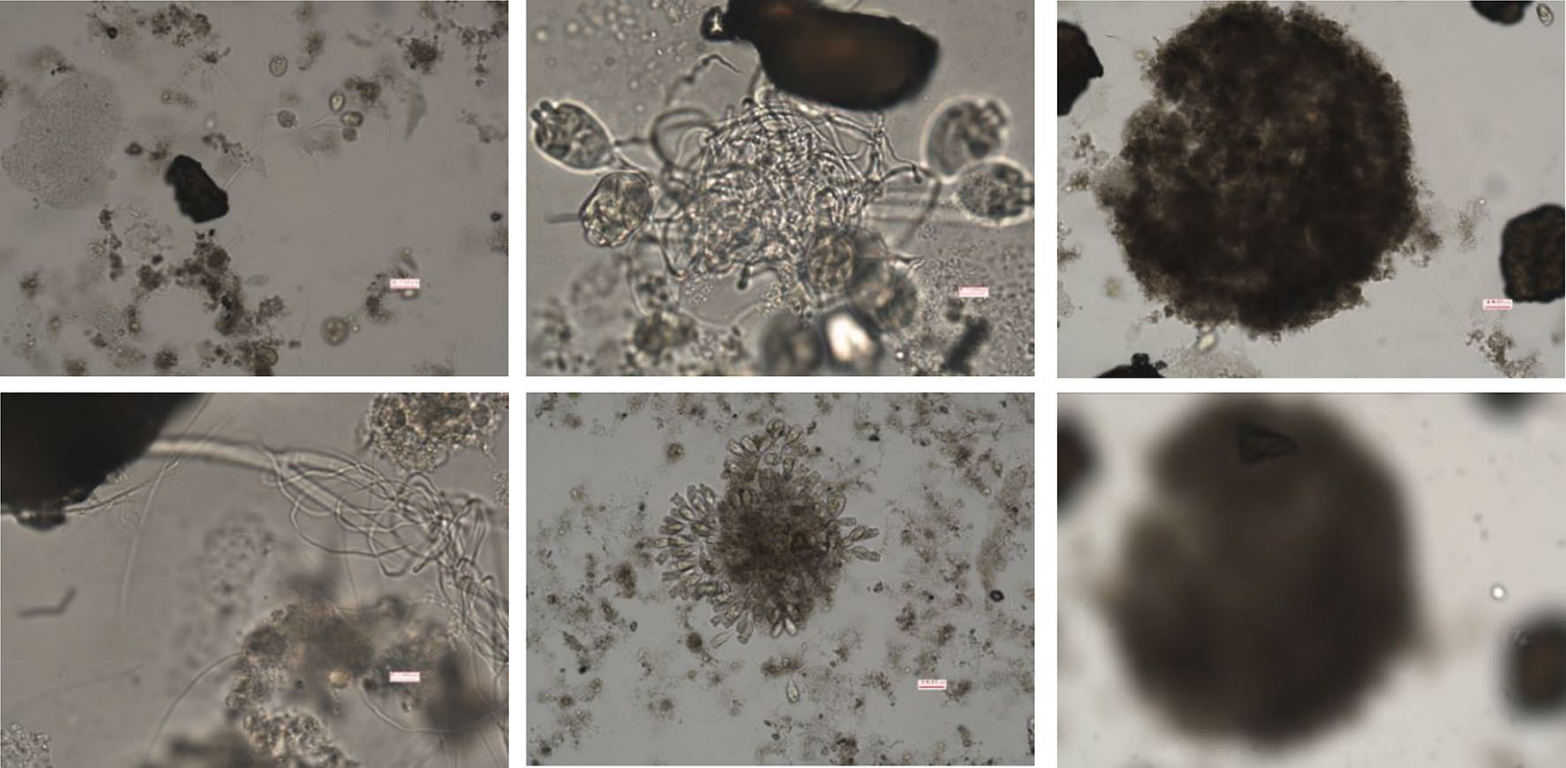
Effect of inert PET-1 particles on filamentous bacteria in activated sludge.

As mentioned earlier, PET-1 particles are easily adsorbed by activated sludge. On the 7th day of this experiment, it was found that many PET-1 particles adsorbed in activated sludge were "captured" by bell worms, indicating that biofilms had grown on the surface of PET particles, as shown in Fig. 4. Under the influence of hydraulic shear forces, filamentous bacteria exhibit the ability to bend and grow, firmly wrapping themselves around the branches of filamentous bacteria. This enables the smaller bacterial flocs to amalgamate into larger bacterial flocs. As shear forces persist and EPS continue to be secreted and accumulate, the bacterial flocs grow in size and density, eventually coalescing into granular bacterial flocs. Figure 4 provides additional visual evidence that the particles are enclosed within the granular bacterial flocs. It is worth mentioning that the PET particles in the remaining two particle grades exhibited higher levels of kinetic energy, a phenomenon that was not observed in the batch test. This observation provides clarifies the role of late-stage PET-1 particles in augmenting the sedimentation rate of activated sludge. This observation provides evidence in favour of the crystal nucleus theory for the development of granular sludge^45^.

### AAO experiment

The processing capacity of the AAO experimental reactor was 1.5 L/h. After 7 days of stable operation, PET particles were added exclusively to Group 1. In contrast, no particles were added to the second group, referred to as the blank group. Based on the findings of the oscillation batch test, the PET-1 particles were chosen for dosing, with an observed abundance of 10,000 particles/L. The system’s particle recharge was determined by assessing the abundance of PET particles and the quantity of sludge discharged to sustain the desired particle count within the activated sludge in the system. Simultaneously, various parameters were assessed, including the concentration of PET-1 particles in the effluent, the characteristics of the incoming and outgoing water, and the SV and SVI of the activated sludge. The microbial properties were studied.

Based on analysis, the concentration of PET particles in the effluent of the device was between 2 and 5/L, demonstrating that a large proportion of the added particles were retained within the AAO system. However, the particle content found within the sludge ultimately stabilized at approximately 4000/L, which was significantly below the intended dosage of 10,000/L. Based on observations, the particles adhered to the system pool wall, the mixing rod, and the sludge. A portion was adsorbed on bubbles and may have exited the system when the bubbles burst, while another part was presumed to sink to the system's bottom.

**Figure 5:**
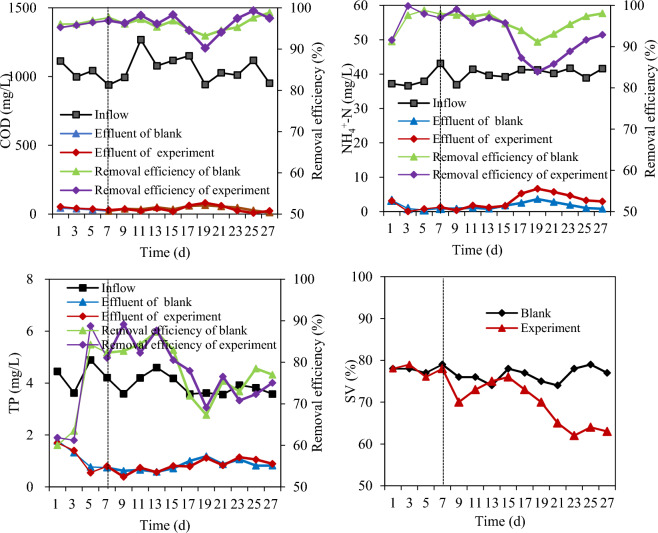
Variations in COD, and NH_4_^+^-N, TP and SV in the AAO.

An analysis of the inlet and exit water quality, as depicted in Fig. 5, reveals that the removal of COD, ammonia nitrogen, and total phosphorus remained stable throughout the test period. Notably, the removal of ammonia nitrogen resulted in an obvious inhibition of nitrification following the introduction of PET particles. This differs from the observed trends reported by Li^46^ and Zhao^47^, who documented that microbes gradually adapted to particulates and increased pollutant removal, particularly COD. Furthermore, after 8 days of PET particle addition, the SV of the sludge began to exhibit a significant decrease, eventually stabilizing at a level between 62 and 65%. This represents an average reduction of 21% compared to the control group, as shown in Fig. 5. Additionally, the SVI displayed a declining trend after the 5th day of particle dosing, ultimately dropping below 200 mg/L. This corresponds to a reduction of approximately 43%, as detailed in Table 3. These findings align with the results reported by Hu et al.^48^ in a sequencing batch reactor (SBR) setup, where the presence of particles in the reactor contributed to a reduction in SVI for normal activated sludge. Table 4 provides a summary of the sludge characteristics for nontreated settled sludge (blank group) and PET-treated sludge (experimental group) after 25 days.

**Table 3:** Results of SVI.

Time (d)	1	5	9	15	21	25
Blank group (mL/g)	358	348	333	381	349	358
Experimental group (mL/g)	349	371	320	313	188	198

**Table 4:** Comparison of sludge characteristics after 25 days.

	MLSS (mg/L)	SV (%)	SVI (mL/g)
Blank group	2150	77	358
Experimental group	3182	63	198

**Figure 6:**
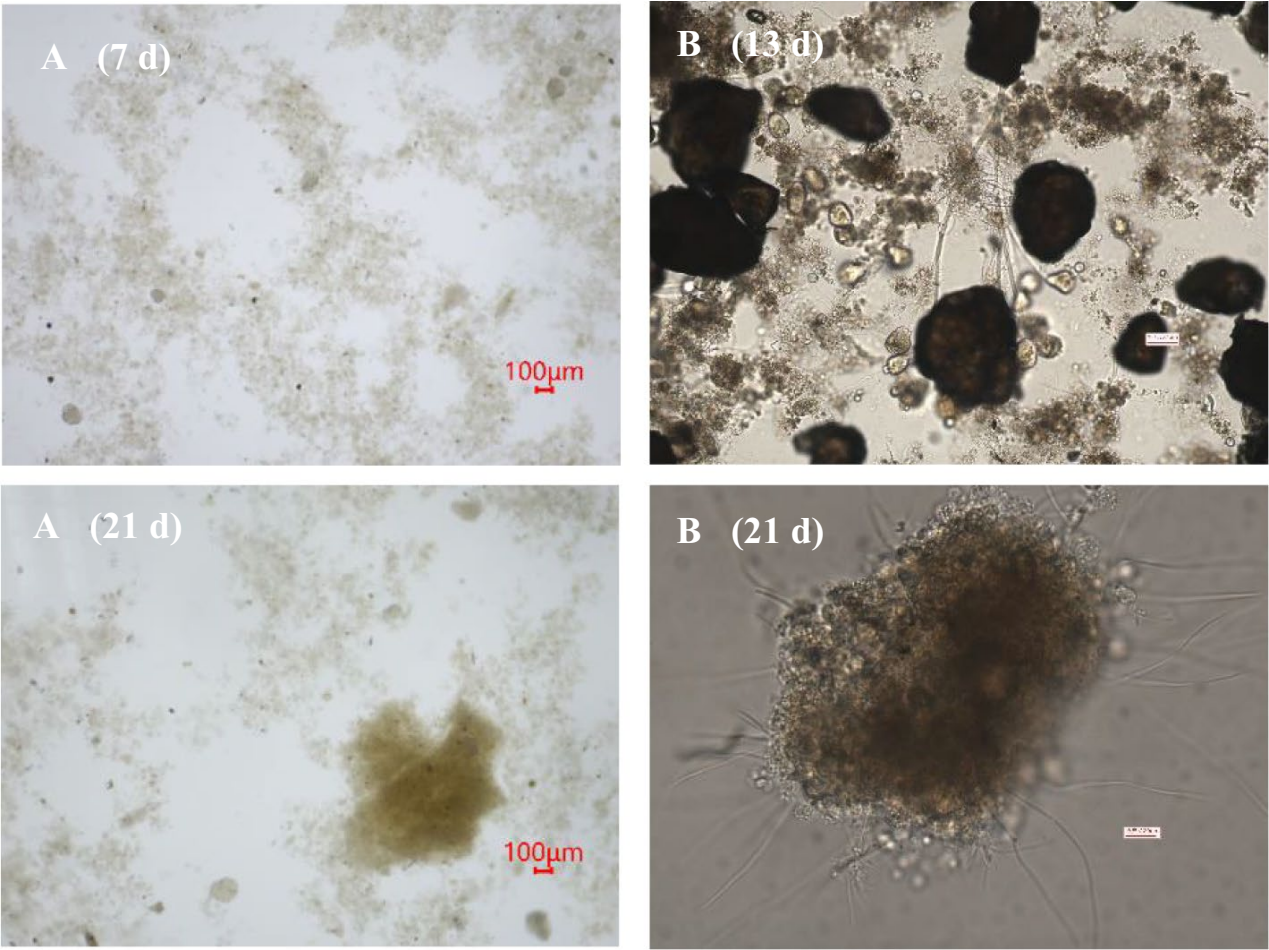
Inert particles in activated sludge.

Microscopy images of particles within the activated sludge are presented in Fig. 6. The stereoscopic observations in Fig. 6 reveal the presence of particles within the bacterial colloidal aggregates. This observation indicates that the improved settling characteristics can be attributed to the addition of particles, which aid in the agglomeration of activated sludge bacterial colloids. However, despite these observations, the SVI test results shown in Table 3 indicate that the settling performance of the activated sludge was suboptimal, and the problem of sludge bulking by filamentous bacteria was not comprehensively resolved. This problem was further underscored by the continued presence of filamentous bacteria in the biofacies, as illustrated in Fig. 6B (21 d).

## Conclusions

This study involved the addition of three different sizes of PET particles to filamentous bacteria-bulked sludge, and the results were compared with a control group with no particle addition. The findings revealed that the addition of particles had a notable impact on both the quantity and growth morphology of filamentous bacteria. Furthermore, it led to a reduction in SVI and an enhancement in the settling performance of the bulked activated sludge. Upon analysing the trends observed in the results, it became evident that larger particle sizes significantly reduced the presence of filamentous bacterial mycelia within the activated sludge bacterial flocs. Conversely, smaller particle sizes appeared to promote the coalescence of bacterial flocs, directly contributing to an increase in the settling rate of activated sludge. Additionally, it was noted that when the particle abundance reached 4000 particles/L, the removal efficiency of the AAO reactor for COD and total phosphorus remained unaltered. However, there was a slight decrease in the removal efficiency of ammonia nitrogen.

## Data Availability

Data are available on request from the corresponding author.
